# *Cryptosporidium parvum* gp40/15 Is Associated with the Parasitophorous Vacuole Membrane and Is a Potential Vaccine Target

**DOI:** 10.3390/microorganisms8030363

**Published:** 2020-03-04

**Authors:** Zhaohui Cui, Luyang Wang, Yuexin Wang, Juan Li, Rongjun Wang, Mingfei Sun, Longxian Zhang

**Affiliations:** 1 College of Animal Science and Veterinary Medicine, Henan Agricultural University, Zhengzhou 450002, China; Cui9008@126.com (Z.C.); wangly0505@gmail.com (L.W.); wangyuexin820@gmail.com (Y.W.); wrj-1978@163.com (R.W.); 2 Key Laboratory of Livestock Disease Prevention of Guangdong Province, Scientific Observation and Experiment Station of Veterinary Drugs and Diagnostic Techniques of Guangdong Province, Ministry of Agriculture, Institute of Animal Health, Guangdong Academy of Agricultural Sciences, Guangzhou 510640, China; lijuan413@126.com

**Keywords:** *Cryptosporidium parvum*, Cpgp40/15, PVM, neutralization, vaccine

## Abstract

*Cryptosporidium parvum* is a zoonotic intracellular protozoan responsible for the diarrheal illness cryptosporidiosis in humans and animals. Although a number of zoite surface proteins are known to be expressed during, and believed to be involved in, attachment and invasion of host cells, the molecular mechanisms by which *C. parvum* invades the host epithelial cells are not well understood. In the present study, we investigated the gene expression patterns, protein localization in developmental stages in culture, and in vitro neutralization characteristics of Cpgp40/15 and Cpgp40. Indirect immunofluorescence assay showed that Cpgp40/15 is associated with the parasitophorous vacuole membrane (PVM) during intracellular development. Both anti-gp40/15 and anti-gp40 antibodies demonstrated the ability to neutralize *C. parvum* infection in vitro. Further studies are needed to fully understand the specific role and functional mechanism of Cpgp40/15 (or gp40/15 complex) in the invasion of the host or in the PVM and to determine the feasibility of gp40/15 as a vaccine candidate for cryptosporidiosis in vivo.

## 1. Introduction

*Cryptosporidium* spp. are important etiological agents of diarrhea and are among the leading causes of moderate to severe diarrhea in children under 2 years [1]. Cryptosporidiosis is self-limiting in immunocompetent hosts but can be a chronic and life-threatening infection in immunocompromised patients [2]. Owing to the significant disease burden in developing countries, the World Health Organization (WHO) has included *Cryptosporidium* in the “Neglected disease initiative” since 2004 [3]. To date, there are no fully efficacious treatment options or vaccines for cryptosporidiosis [4]. Although nitazoxanide is approved for treatment of cryptosporidiosis in immunocompetent individuals, it has not been approved for use by immunocompromised patients [5].

Currently, the mechanisms that contribute to disease caused by *Cryptosporidium* are not fully understood [6]. Several putative *Cryptosporidium*-specific virulence factors have been made to identify and characterize proteins involved in the initial interactions between the pathogens and host cells [7,8]. The initial interaction processes of *Cryptosporidium* oocysts and sporozoites with host epithelial cells can be divided into several major developmental phases: excystation, gliding motility, attachment, invasion, parasitophorous vacuole formation, intracellular maintenance, and host cell damage [9,10]. *Cryptosporidium* does not normally cause systemic infection or penetrate deep tissue; rather, the parasite establishes itself in a membrane-bound compartment, termed the parasitophorous vacuole (PV), on the apical surface of the intestinal epithelium [11]. Additionally, the host cell-derived parasitophorous vacuole membrane (PVM) structure separates the intracellular parasites from the host cell cytosol [12].

Cpgp40/15 (also referred to as gp60) was first described by Strong [13] and Cevallos [14] and is a sporozoite and merozoite cell surface protein. The gp40/15 mRNA is translated into a 60-kDa glycoprotein precursor during the intracellular stages of the *Cryptosporidium parvum* life cycle and is proteolytically processed to generate 15- and 45-kDa glycoproteins after synthesis [15]. Both gp40 and gp15 display O-linked α-N-acetylgalactosamine (α-GalNAc), which is thought to be involved in invasion and attachment [16]. Nevertheless, gp40 and gp15 seemed to associate after proteolytic cleavage to generate a protein complex capable of linking zoite and host cell surfaces [17]. Different biological functions of gp40 and gp15, as well as the precursor protein gp40/15 (or gp40/15 complex) may play an important role in the host–parasite interaction. In addition, subtyping tools targeting the gp60 gene have been used extensively in assessing the intraspecies diversity of *C. parvum*, *C. hominis*, and other human-pathogenic *Cryptosporidium* spp., indicating significant phenotypic differences between subtype families [18].

In the present study, we investigated the gene expression patterns, protein localization in developmental stages in culture, and in vitro neutralization characteristics of Cpgp40/15 and Cpgp40 to gain deeper insights into the biological role of Cpgp40/15 in *C. parvum*.

## 2. Materials and Methods

### 2.1. Ethics Statement

The animal handling and experimental procedures were carried out in compliance with recommendations of the Guide for the Care and Use of Laboratory Animals of the Ministry of Health, China. The experimental protocol was approved by the Institutional Animal Care and Use Committee of Henan Agricultural University on 10 July 2015 (authorization number IACUC-henau-20150710).

### 2.2. Parasite and Cell Lines

*C. parvum* (Iowa isolate) oocysts were purchased from Waterborne, Inc. (New Orleans, LA, USA) and stored in phosphate-buffered saline (PBS) at 4 °C for up to 3 months (from harvest) before use. Before experiments, oocysts were treated with 10% Clorox on ice for 10 min and washed three times with sterile PBS. Free sporozoites were prepared by incubating oocysts in PBS containing 0.25% trypsin and 0.75% taurodeoxycholic acid at 37 °C for 2 h. Human ileocecal adenocarcinoma (HCT-8) cells (American Type Culture Collection, Manassas, VA, USA) were cultured and maintained in Dulbecco’s modified Eagle’s medium (DMEM) supplemented with 10% fetal bovine serum, 100 U/mL penicillin, and 100 U/mL streptomycin at 37 °C in a humidified 5% CO_2_ incubator. For in vitro experiments, HCT-8 cells were transferred to 12-well cell culture plates and monolayers grown to 80–90% confluence. *C. parvum* oocysts were added into the cell culture at a parasite:host cell ratio of 1:5 (i.e., 2 × 10^5^ oocysts/well). After incubation at 37 °C for 3 h that allowed sporozoites invade host cells, uninvaded parasites were removed by a medium exchange. Intracellular parasites were allowed to grow for specified times before subsequent experiments including RNA isolation for gene expression analysis or fixation for immunofluorescence staining.

### 2.3. Cpgp40/15 and Cpgp40 Cloning, Expression, and Purification

The following two fragments were amplified by PCR from *C. parvum* (Iowa) genomic DNA with the following primers (the added restriction sites are underlined): a 903-bp fragment encoding 294 amino acids (corresponding to the entire Cpgp40/15 ORF (open reading frame), minus the putative signal peptide), forward, 5′-CGCGAATTCGATGTTCCTGTTGAGGGCTC-3′; reverse, 5′-CGCGTCGACCAACACGAATAAGGCTGC-3′. A 588-bp fragment encoding 190 amino acids (corresponding to Cpgp40), forward, 5′-CGCGAATTCGATGTTCCTGTTGAGGGCTC-3′; reverse, 5′-CGCGTCGACCTCTGAGAGTGATCTTCTTG-3′. PCR amplification was performed under the following conditions: denaturation at 95 °C for 5 min, 35 cycles of amplification at 95 °C for 45 s, 57 °C for 45 s, and 72 °C for 1 min; and a final extension at 72 °C for 10 min. PCR products were purified using a TIANgel Midi Purification Kit (TIANGEN Biotech, Beijing, China), digested with EcoRI and SalI restriction enzymes (New England Biolabs, Beijing, China), and inserted into the expression vector pGEX-4T-1 (Novagen, Madison, WI, USA). *Escherichia coli* DH5α cells (TIANGEN Biotech, Beijing, China) were transformed with the ligation products and grown on Luria–Bertani (LB) agar plates with 50 μg/mL ampicillin, with positive colonies being identified by PCR and sequencing. *E. coli* Rosetta (DE3) cells (TIANGEN Biotech) were transformed with the recombinant plasmids and cultured in LB medium supplemented with 50 μg/mL ampicillin. The induction of *Cpgp40/15* and *Cpgp40* expression was performed by adding 0.1 mM isopropyl-β-D-thiogalactopyranoside (IPTG) at 37 °C for 3 h. The expression levels and solubility of the target proteins were evaluated by using SDS-PAGE with Coomassie blue G-250 staining.

For the purification of Cpgp40/15 and Cpgp40, cultured *E. coli* were collected by centrifugation, re-suspended in PBS buffer, and disrupted by sonication on ice. The lysate was centrifuged and the supernatant was filtered through a 0.45 μm cellulose acetate membrane filter (Millipore, Billerica, MA, USA) and loaded onto Glutathione Sepharose 4B beads (GE Healthcare, Pittsburgh, USA) at 4 °C and 90 rpm for 3 h. After washing the beads with six volumes of PBS, Cpgp40/15 and Cpgp40 were eluted from the beads with elution buffer (50 mM Tris-HCl, 10 mM reduced glutathione, pH 8.0). The purified proteins were examined by SDS-PAGE on a 10% gel.

### 2.4. Preparation of Cpgp40/15 and Cpgp40 Polyclonal Antibodies

Polyclonal antibodies against Cpgp40/15 and Cpgp40 were raised in pathogen-free rabbits by Sangon Biotech (Shanghai, China). Primary immunization was conducted on days 1 and 21 using 300 μg of purified Cpgp40/15 and Cpgp40 protein emulsified in an equal volume of Freund’s complete adjuvant. Immunized animals received boost immunizations four times every seven days with 150 μg of Cpgp40/15 and Cpgp40 protein in Freund’s incomplete adjuvant. Seven days after the final immunization, rabbit sera were collected, and the polyclonal IgG antibodies were purified from the immune sera using protein A Sepharose affinity chromatography. The titer and specificity of the antibodies were evaluated using an enzyme-linked immunosorbent assay (ELISA) and Western blot, respectively.

### 2.5. Western Blot Analysis of Native Cpgp40/15 and Cpgp40

For Western blot analysis of the native Cpgp40/15 and Cpgp40, oocysts treated with 10% Clorox were suspended in PBS buffer containing 0.75% taurodeoxycholic acid and 0.25% trypsin and incubated at 37 °C for 3 h. The released sporozoites were collected by centrifugation and resuspended in cell lysis buffer containing 1% protease inhibitor cocktail (Solarbio, Beijing, China). Similarly, HCT-8 cells (≈2 × 10^6^) were lysed in the same way. The proteins (from ≈2 × 10^7^ sporozoites/lane and ≈5 × 10^5^ HCT-8 cells/lane) were separated by SDS-PAGE and transferred onto a polyvinylidene fluoride (PVDF) membrane using a semi-dry electro-blotting apparatus (Bio-Rad, Hercules, CA, USA) running at 300 mA for 2 h. After blocking with PBST containing 5% bovine serum albumin (BSA) at room temperature (RT) for 1 h, the membrane was incubated overnight with anti-Cpgp40/15 antibodies (≈1.3 μg/mL), anti-Cpgp40 antibodies (≈1.3 μg/mL), or pre-immune serum (1:500). The following day, the PVDF membrane was washed three times with PBST and incubated at RT with 1:2000 horseradish peroxidase (HRP)-conjugated goat anti-rabbit IgG (H + L) (Proteintech, Wuhan, China) for 1 h. Finally, the membrane was washed three times with PBST and reactive protein bands in the membrane were detected using the Immobilon Crescendo Western HRP substrate (Merck Millipore, MA, USA) and analyzed with an Amersham Imager 680 (GE, CT, USA).

### 2.6. Indirect Immunofluorescence Microscopy

Sporozoites resuspended in PBS were dried onto microscope slides, while the intracellular stages of *C. parvum* in HCT-8 cell were grown on coverslips for 9, 12, and 18 h. The slides and coverslips were fixed at RT for 20 min with 4% paraformaldehyde. After three washes in PBS, the fixed cells were permeabilized with 0.5% Triton X-100 in PBS for 15 min, washed three times with PBS, blocked with 5% BSA in PBS (BSA-PBS) at RT for 30 min, and incubated overnight with anti-Cpgp40/15 and anti-Cpgp40 antibodies (≈1.3 μg/mL) in 5% BSA-PBS, respectively. In addition, the slides and coverslips treated as above and incubated overnight with pre-immune serum served as controls. After three washes in PBS, the cells were incubated with Alexa Fluor^®^ 594-conjugated goat anti-rabbit IgG (Bioss, Beijing, China) in BSA-PBS at 1:500 for 1 h. After three washes with PBS, the cells were counter stained with the nuclear stain 4′, 6-diamidino-2-phenylindole (DAPI, Yeasen, Shanghai, China). After another three washes with PBS, the slides and coverslips were mounted with No-Fade Mounting Medium (Yeasen, Shanghai, China) and examined by differential interference contrast (DIC) and fluorescence microscopy using a LSM 710 laser confocal microscope (Zeiss, Jena, Germany).

### 2.7. Examination of Cpgp40/15 Expression by qPCR

The relative expression levels of the *Cpgp40/15* gene in intracellular parasites in HCT-8 cultures at 0–48 h (3, 6, 9, 12, 24, and 48 h post-infection) was evaluated by qPCR. The expression of the 18S rRNA gene was determined in parallel for data normalization [19]. Using HiPure Total RNA Plus Kits (Magen, Guangzhou, China), total RNA was extracted from *C. parvum*-infected HCT-8 cells at each culture point. Then, first-strand cDNA was synthesized using the PrimeScript™ RT Reagent Kit with gDNA Eraser (TaKaRa, Dalian, China). qPCR was performed in a 25 μL reaction volume containing 2 μL (approximately 100 ng) of cDNA, 12.5 μL of 2× SYBR1 Premix Ex TaqTM II (Tli RNaseH Plus, TaKaRa), 0.5 μL each of the forward and reverse primers (10 μM), and 7 μL of deionized water in a CFX384™ Real-Time PCR system (Bio-Rad, Hercules, CA, USA). The primers used included 5′-GATTTGTTTGCCTTTACCCT-3′ and 5′-CCAAGTCTCCGTTCTCATTC-3′ for the *Cpgp40/15* gene and 5′-TAGAGATTGGAGGTTGTTCCT-3′ and 5′-CTCCAC CAACTAAGAACGGCC-3′ for 18S rRNA. The relative expression of the *Cpgp40/15* gene was calculated using the 2^−ΔΔCT^ method [20]. The data are presented as the means ± standard error of the mean (SEM).

### 2.8. In Vitro Neutralization of Sporozoite Invasion

The effect of polyclonal anti-Cpgp40/15 and anti-Cpgp40 antibodies on *C. parvum* infection of HCT-8 cells were examined using an in vitro neutralization assay. Briefly, HCT-8 cells were grown in 12-well plates to 80–90% confluence and maintained in DMEM supplemented with 10% FBS, 100 U/mL penicillin, and 100 U/mL streptomycin at 37 °C in a humidified 5% CO_2_ incubator. Oocysts were treated with 10% Clorox on ice for 10 min and washed three times with cold sterile PBS. For neutralization assays, 2 × 10^5^ oocysts were incubated with different dilutions of antibodies or pre-immune serum in infection medium in HCT-8 cell culture at 37 °C for 2 h. Based on results of preliminary evaluations, 1:50 (≈13 μg), 1:100 (≈6.5 μg), and 1:500 (≈1.3 μg) dilutions of antibodies were used in neutralization assays. After 3 h incubation, free sporozoites were washed off and incubated for an additional 24 h. The method for assessment of antibodies against *C. parvum* infection of HCT-8 cells in vitro was based on the quantitative real-time reverse transcription-PCR (qRT-PCR) technique, as described in Cai et al. [19].

## 3. Results

### 3.1. Expression of Recombinant Cpgp40/15 and Cpgp40

**Figure 1:**
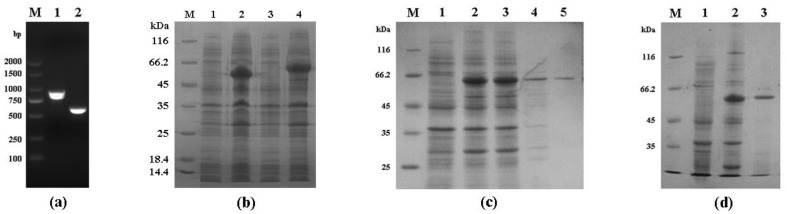
Expression of Cpgp40/15 and Cpgp40 in *Escherichia coli*. (**a**) PCR amplification of the *Cpgp40/15* and *Cpgp40* genes of *Cryptosporidium parvum*. Lane M: 2000 bp DNA ladder maker. Lane 1: Cpgp40/15 product, Lane 2: Cpgp40 product. (**b**) SDS-PAGE analysis of Cpgp40/15 and Cpgp40 expressed in *E. coli*. Lane M: molecular weight markers. Lanes 1,3: lysate from bacterial cells transformed with pGEX-4t-1-Cpgp40/15 or pGEX-4t-1-Cpgp40 without isopropyl-β-D-thiogalactopyranoside (IPTG) induction, respectively. Lanes 2,4: lysate from bacterial cells transformed with pGEX-4t-1-Cpgp40/15 or pGEX-4t-1-Cpgp40 with IPTG induction, respectively. (**c**) Purity of recombinant Cpgp40/15 assessed by SDS-PAGE analysis. Lane M: molecular weight markers, Lane 1: lysate from recombinant bacterial cells without IPTG induction. Lane 2: lysate from recombinant bacterial cells with IPTG induction. Lane 3: supernatant of IPTG-induced recombinant bacterial cells. Lane 4: sediment of lysate from IPTG-induced recombinant bacteria cells. Lane 5: Cpgp40/15 purified using Glutathione Sepharose affinity chromatography. (**d**) Purity of recombinant Cpgp40 assessed by SDS-PAGE analysis. Lane M: molecular weight markers, Lane 1: lysate from recombinant bacterial cells without IPTG induction. Lane 2: supernatant of IPTG-induced recombinant bacterial cells. Lane 3: Cpgp40 purified using Glutathione Sepharose affinity chromatography.

**Figure 2:**
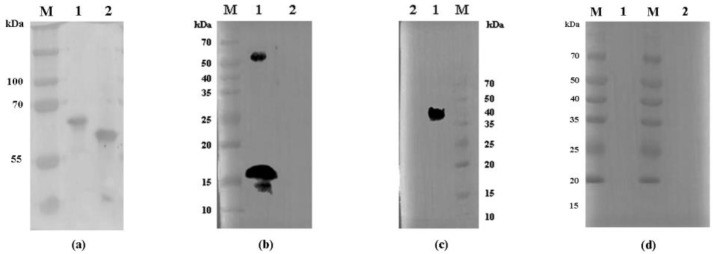
Western blot analysis of recombinant Cpgp40/15 and Cpgp40 in *E. coli* and native Cpgp40/15 and Cpgp40 in *C. parvum* sporozoites. (**a**) Expression of recombinant Cpgp40/15 and Cpgp40 in *E. coli* assessed by Western blot analysis using an anti-GST tag. Lane M: protein size makers. Lanes 1,2: lysate from bacterial cells transformed with pGEX-4t-1-Cpgp40/15 or pGEX-4t-1-Cpgp40 with IPTG induction, respectively. (**b**) Western blot analysis of native Cpgp40/15 in *C. parvum* sporozoites. Lane M: protein size makers. Lane 1: native proteins from sporozoites. Lane 2: proteins from human ileocecal adenocarcinoma (HCT-8) cells. (**c**) Western blot analysis of native Cpgp40 in *C. parvum* sporozoites. Lane M: protein size makers. Lane 1: native proteins from sporozoites. Lane 2: proteins from HCT-8 cells. (**d**) Western blot analysis of native Cpgp40/15 and Cpgp40 using pre-immune serum. Lane M: protein size makers. Lanes 1,2: native proteins from sporozoites.

The *Cpgp40/15* gene was successfully cloned (Figure 1a) and expressed in *E. coli* Rosetta (DE3) cells (Figure 1b). SDS-PAGE analysis revealed the molecular masses of recombinant Cpgp40/15 and Cpgp40 to be a little larger than the expected sizes of ≈56 and ≈46 kDa. Recombinant Cpgp40/15 and Cpgp40 were also confirmed by Western blot analysis using anti-GST tag antibodies (Figure 2a). Recombinant Cpgp40/15 and Cpgp40 were then purified with a GST-tag, and the target proteins were eluted by using 10 mM reduced glutathione (Figure 1c,d).

### 3.2. Identification of Native Cpgp40/15 and Cpgp40

Antibodies against recombinant Cpgp40/15 and Cpgp40 were generated to characterize native Cpgp40/15 and Cpgp40. Antibodies against Cpgp40/15 recognized a protein of ≈50 kDa and a band of ≈15 kDa in lysates of *C. parvum* sporozoites (Figure 2b). Antibodies against Cpgp40 reacted with a protein of ≈40 kDa in lysates of *C. parvum* sporozoites (Figure 2c). Neither antibody reacted with endogenous HCT-8 cell proteins (Figure 2b,c). Furthermore, the pre-immune serum did not react with any proteins from lysates of *C. parvum* sporozoites (Figure 2d).

### 3.3. Cpgp40/15 Is Associated with the PVM during Intracellular Development

**Figure 3:**
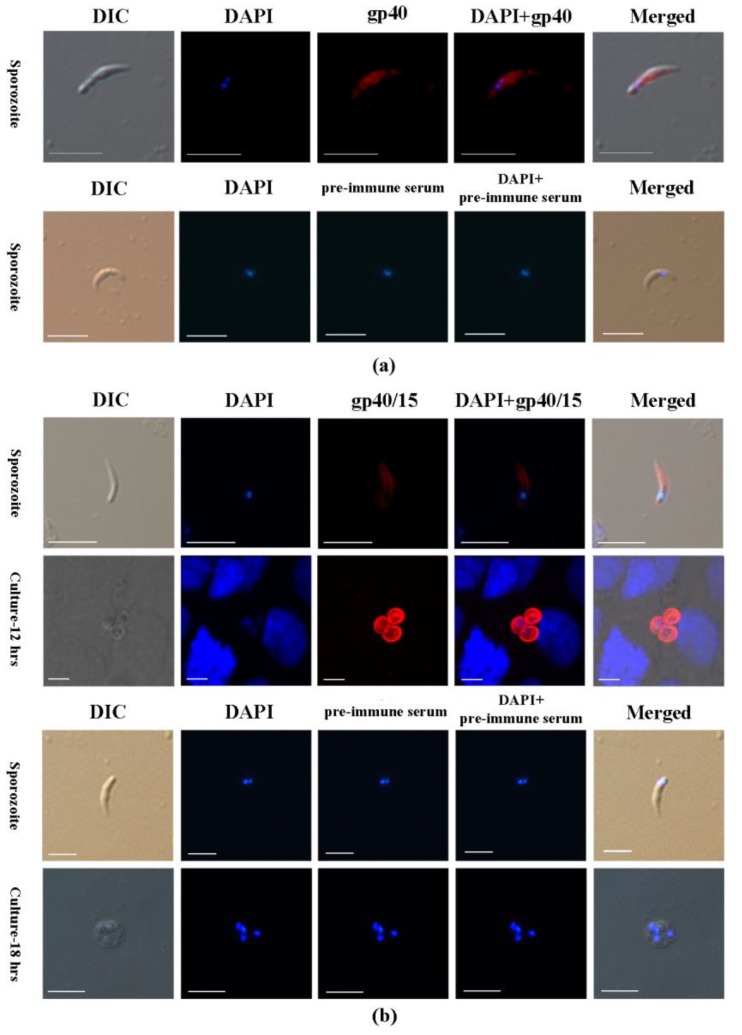
Immunofluorescence microscopic detection of Cpgp40/15 and Cpgp40 in different *C. parvum* life cycle stages. (**a**) Distribution of Cpgp40 in the sporozoites using the rabbit anti-Cpgp40 antibody and pre-immune serum. (**b**) Association of Cpgp40/15 with the parasitophorous vacuole membrane (PVM) during the parasite intracellular developmental stages, including early and mature meronts, using rabbit anti-Cpgp40/15 antibody and pre-immune serum. Images were taken by differential interference contrast microscopy (DIC), fluorescence microscopy using nuclear stain 4,6-diamidino-2-phenylindole (DAPI); Scale-bars: 5 μm.

The distribution of Cpgp40/15 and Cpgp40 on sporozoites and developmental stages of *C. parvum* in HCT-8 cell cultures at 9, 12, and 18 h were examined by immunofluorescence. Both anti-Cpgp40/15 and anti-Cpgp40 antibodies reacted with the surface of free sporozoites (Figure 3a,b) when observed by immunofluorescence microscopy. However, during parasite intracellular development (at 12 h of cell culture), the Cpgp40/15 antibodies had a high reactivity to the PVM (Figure 3b). No signals were observed in parasites or in host cells when using pre-immune sera (Figure 3a,b). In addition, no significant signals were observed in parasites or in host cells during the intracellular stages of infection when using anti-Cpgp40 polyclonal antibodies.

### 3.4. Cpgp40/15 Is Expressed throughout Intracellular Development

**Figure 4:**
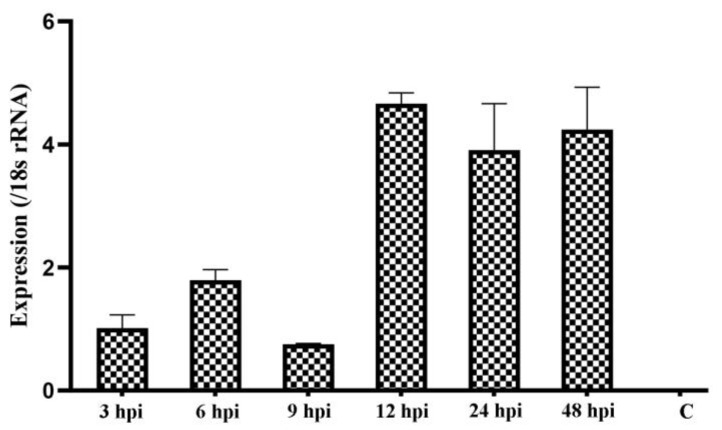
Relative expression of the *Cpgp40/15* gene in HCT-8 cell culture of *C. parvum* after the normalization of Ct values of qPCR with data from the 18S rRNA gene of *C. parvum*. Data shown are mean ± SD from three replicate assays.

We examined the transcript expression of the *Cpgp40/15* gene during parasite development in vitro based on qPCR analysis. The data were normalized using Ct values of the 18S rRNA gene of *C. parvum*. The expression of gp40/15 was assessed over a 48 h time course in *C. parvum*-infected HCT-8 cells. The highest expression levels were detected at 12 hpi (Figure 4).

### 3.5. Cpgp40/15 and Cpgp40 Antibodies Inhibit Cryptosporidium Infection In Vitro

**Figure 5:**
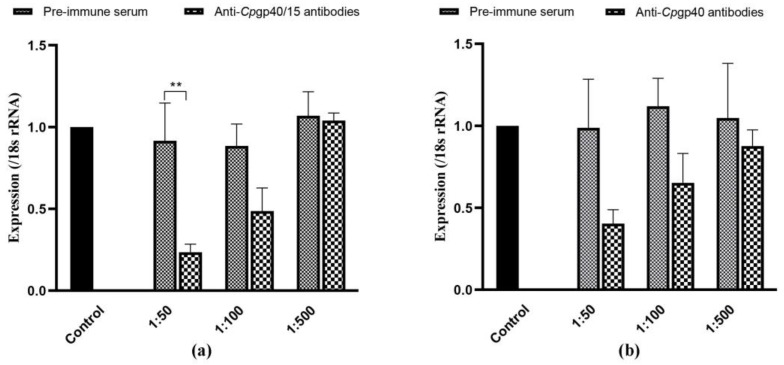
Inhibition of *Cryptosporidium parvum* invasion of HCT-8 cells by polyclonal Cpgp40/15 and Cpgp40 antibodies. (**a**,**b**) Control, *C. parvum* culture maintained in growth medium alone. 1:50, *C. parvum* culture with the addition of pre-immune or immune antibodies diluted 1:50. 1:100, *C. parvum* culture with the addition of pre-immune or immune antibodies diluted 1:100; 1:500, *C. parvum* culture with the addition of pre-immune or immune antibodies diluted 1:500; data shown are mean ± SD from three replicate assays.

The effect of polyclonal anti-Cpgp40/15 and anti-Cpgp40 antibodies on *C. parvum* infection was evaluated by an invasion neutralization assay. Comparable parasite loads, as measured by qRT-PCR, between the control cultures and parasite cultures incubated with 1:50, 1:100, and 1:500 dilutions of the pre-immune serum were obtained 24 h after invasion. When compared with the control culture, we found that the parasite load was reduced significantly when the cell culture was inoculated with sporozoites treated with antibodies against Cpgp40/15 (*p* < 0.01) and Cpgp40 at 1:50 dilutions (Figure 5a,b).

## 4. Discussion

Cpgp40/15, the best characterized of the zoite antigens, is a mucin-like glycoprotein antigen that is synthesized as a single precursor protein and proteolytically cleaved into two surface glycoproteins, gp40 and gp15 [21]. The Cpgp40/15 gene does not contain introns and is present in a single copy in the *C. parvum* genome [13]. Analysis of the Cpgp40/15 ORF revealed the presence of an N-terminal signal peptide, a polyserine domain, multiple predicted O-glycosylation sites, a single potential N-glycosylation site, and a hydrophobic region at the C terminus, a finding consistent with what is required for the addition of a GPI anchor [14]. Previous studies have reported that gp40/15 mRNA is translated into a 49-kDa glycoprotein precursor during the intracellular stages of the *C. parvum* life cycle, while gp40 has been thought to bind intestinal epithelial cells by recognizing a host cell receptor [14]. In the present study, we cloned and expressed the fragments of Cpgp40/15 (corresponding to the entire Cpgp40/15 ORF) and Cpgp40 (corresponding to the entire Cpgp40) in *E. coli* in order to investigate the functions of these antigens.

In previous studies, native gp40 and gp15 were present in the oocysts, sporozoites, and shed proteins by immunoblotting analysis with anti-gp40 antisera and MAb CrA1/2, while the precursor protein gp40/15 was expressed by intracellular stages of the parasite [14,17]. Both gp40 and gp15 were localized to the membrane of sporozoites (gp40 was also found in the apical region of the sporozoite), inner surface of the oocyst wall, and were shed in trails during gliding motility [13,14,17,22]. Moreover, the two proteins were also present on the merozoite membrane during the intracellular stages, and this was thought to be due to co-localization of gp40 and gp15 (or a gp40/15 complex) [17]. In the present study, we performed an indirect immunofluorescence assay on *C. parvum* sporozoites and found that both anti-gp40/15 and anti-gp40 antibodies reacted with the surface of live sporozoites. Furthermore, fluorescence signals were also observed in the PVM during parasite intracellular development using anti-Cpgp40/15 antibodies. It is worth mentioning that the anti-Cpgp40/15 antibodies recognized a protein of ≈50 kDa (native Cpgp40/15) in lysates of *C. parvum* sporozoites. The Cpgp40/15 precursor was also recognized in Caco-2A cells infected with *C. parvum* for 18 h [14]. Biosynthetic labeling experiments using intracellular parasites have shown that the Cpgp40/15 precursor is rapidly cleaved [21], and the co-localization of gp40 and gp15 may be attributable to the gp40/gp15 complex being shed off the sporozoite surface during excystation or is regulated by an equilibrium [17]. However, Cpgp40/15 seemed to be associated with the PVM, either as a precursor or gp40/gp15 complex. Currently, the protein composition, formation mechanism, and function of the cryptosporidial PVM is poorly understood. Only a few proteins involved in fatty acid metabolism and lactate fermentation have been localized to the PVM, such as CpACBP, CpORP1, CpLCE, and CpLDH [12,23,24]. The specific role and functional mechanism of Cpgp40/15 (or gp40/15 complex) in the invasion of the host or in the PVM requires further investigation.

From the published data in CryptoDB (https://cryptodb.org/cryptodb/), Cpgp40/15 transcripts were elucidated in *C. parvum* oocysts, sporozoites, and intracellular stages by RT-PCR and RNA-seq analysis [25,26,27,28,29,30]. In general, the level of Cpgp40/15 transcripts was relatively low in oocysts, sporozoites and during early intracellular development (from 2 to 6 h post-infection) [25,26,27,28,29,30]. The highest expression level of Cpgp40/15 transcripts was detected at 12 hpi over a 72 h infection within HCT-8 cells using RT-PCR, but gradually decreased with the time of infection [25]. Similarly, in the present study, qRT-PCR showed that Cpgp40/15 was expressed throughout the examined time course and was also expressed at low level early in the infection (from 3 to 9 h post-infection) with a peak in transcript levels at 12 hpi. However, the expression level was relatively high at 24 and 48 hpi in the present study.

Evasion of the immune system is the key strategy for successful host invasion by *C. parvum*, which is accomplished by stage-specific expression of unique antigens [31]. Antibodies developed against some of these antigens have had therapeutic effectiveness in vitro or experimental animal models [32]. Neutralizing antibodies may take effect by blocking the parasite’s interaction with the host cell by preventing attachment and/or invasion, or binding to sporozoite surfaces, which may interfere with development, resulting in an aborted infection or the production of defective oocysts [33]. So far, over 30 *Cryptosporidium* antigens have been identified in the early interactions between the pathogens and host cells, either as surface proteins or as part of the apical complex [8]. CP15, which is located on sporozoite surface, could stimulate antibody production capable of neutralizing parasite entry to in vitro culture cells [34]. The monoclonal antibodies directed to circumsporozoite-like (CSL) protein were shown to completely neutralize sporozoite infectivity in vitro and limit in vivo infection in a mouse model [35]. Moreover, *Cryptosporidium* calcium-dependent protein kinase 1 (CDPK1) and *Plasmodium* lipid kinase PI(4)K have been identified as attractive targets for treatment, and inhibitors have been developed which inhibit CDPK1 or PI(4)K and are active against *Cryptosporidium* growth both in vitro and in vivo [36,37]. In the present study, both anti-gp40/15 and anti-gp40 antibodies demonstrated the ability to neutralize *C. parvum* infection in vitro, which indicates that Cpgp40/15 may be a candidate for vaccines against cryptosporidiosis.

## 5. Conclusions

In this study, we expressed and characterized the surface proteins Cpgp40/15 and Cpgp40 of *C. parvum*. Compared to previous studies, indirect immunofluorescence assay indicated that Cpgp40/15 is associated with the PVM during intracellular development. Both anti-gp40/15 and anti-gp40 antibodies showed the ability to neutralize *C. parvum* infection in vitro. Further studies are needed to fully understand the specific role and functional mechanism of Cpgp40/15 (or gp40/15 complex) in the invasion of the host or in the PVM and to determine the feasibility of gp40/gp15 as a vaccine candidate for cryptosporidiosis in vivo.
